# Anxiety in Children Undergoing VCUG: Sedation or No Sedation?

**DOI:** 10.1155/2008/498614

**Published:** 2008-07-03

**Authors:** David W. Herd

**Affiliations:** Department of Paediatrics, Starship Children's Hospital, University of Auckland, Private Bag 92024, Auckland, New Zealand

## Abstract

*Background*. Voiding cystourethrograms are distressing for children and parents. Nonpharmacological methods reduce distress. Pharmacological interventions for VCUG focus on sedation as well as analgesia, anxiolysis, and amnesia. Sedation has cost, time, and safety issues. Which agents and route should we use? Are we sure that sedation does not influence the ability to diagnose vesicoureteric reflux? *Methods*. Literature search of Medline, EMBASE, and the Cochrane Database. Review of comparative studies found. *Results*. Seven comparative studies including two randomised controlled trials were reviewed. Midazolam given orally (0.5-0.6 mg/kg) or intranasally (0.2 mg/kg) is effective with no apparent effect on voiding dynamics. Insufficient evidence to recommend other sedating agents was found. Deeper sedating agents may interfere with voiding dynamics. *Conclusion*. Midazolam reduces the VCUG distress, causes amnesia, and does not appear to interfere with voiding dynamics. Midazolam combined with simple analgesia is an effective method to reduce distress to children undergoing VCUG.

## 1. INTRODUCTION

The voiding cystourethrogram (VCUG) is the gold standard for diagnosing vesicoureteric reflux (VUR) and a number of other bladder conditions [1, 2]. The VCUG is a procedure performed mainly on infants and young children in the Radiology Department [3]. There is increasing belief that interventions for VUR are less effective than anticipated, but much debate remains [4–11]. The child is required to be conscious, a urinary catheter is inserted, and the bladder is filled with radio-opaque material, then the child is asked to spontaneously void [12]. This procedure creates distress in the child, the parents, and occasionally staff [13–15]. Nonpharmacological methods to reduce this distress include education prior to the procedure, distraction during, and rewards after [14, 16–28].

Pharmacological interventions primarily focus on sedation but also could include beneficial analgesic, anxiolytic, and amnesic effects [29, 30]. Sedation brings with it cost, time, and safety concerns [1, 29–33]. It is unknown whether we can predict which children will go on to have distress or whether we should sedate routinely [34]. Which agents should we use, and what is the best route of administration? The majority of children having VCUG would not have had one previously. Coping styles and parent-child interaction are important determinants of distress during a medical procedure [34]. Safety of sedating agents is excellent in the context of a sedation service with the necessary staff and equipment to manage sedation in young children [35–37]. Those who sedate children should be prepared for inadvertent deeper sedation, basic life support, and airway management [29–31, 38–40]. Advanced help should be available. Time and cost factors limit the introduction of this distress-reducing intervention. Sedation recovery area and staff time are being the primary cost factors. The medications themselves are relatively inexpensive. Finally, are we sure that sedation does not influence the VCUGs ability to diagnose vesicoureteric reflux?

## 2. METHODS

These methods include a sensitive search of PubMed (1950–2007), EMBASE (1980–2007), Cochrane Database of Systematic Reviews, and Cochrane Randomised Controlled Trials Register. Articles on VCUG were identified through the terms urography (MESH heading exploded), micturating, or voiding cystourethrogram using wildcard search for variations of spelling. Acronyms VCUG and MCUG were also used. To identify sedation articles, the following exploded MESH terms were used: “hyponotics and sedatives,” conscious sedation, midazolam, propofol, chloral hydrate, and nitrous oxide. “Sedation” was searched for as a title word. Results of the VCUG search and sedation search were combined. There were no limits on language for search, but only English language articles were reviewed. Further studies were identified from bibliographies. Unpublished studies were not actively sought.

## 3. RESULTS

Medline search (2008) found 234 papers of which 17 were considered to be of interest [6, 8, 13, 14, 16, 41–52]. EMBASE search found 416 papers of which additional 8 papers were of interest [27, 35, 53–59]. Cochrane Randomised Controlled Trials Register found no further articles of interest. Cochrane Database of Systematic Reviews found one review on interventions for primary vesicoureteric reflux, but none on sedation for this procedure [7]. A review of the bibliographies identified** further 39 papers of interest [2–5, 9, 11, 15, 17–22, 26, 28, 34, 37, 40, 60–81]. Four papers on anesthesiology for VCUG were found and included for discussion [82–85].

Any study comparing a sedative against another sedative, placebo, or standard treatment for VCUG was reviewed. One French language article [77] and one Polish language article [56] were not included. Study designs are summarised in Table 1. Outcome measures and results are in Table 2 [41, 43, 45, 47, 52, 59]. Quality assessment is shown in Table 3. Only two of the studies [45, 52] were of high quality with Jadad scores [86] of 4 or more. One unpublished comparative study was found, but not included [68].

## 4. DISCUSSION

The best way to avoid the distress of the VCUG is not to do the procedure. A better way to image vesicoureteric reflux has recently been discussed in an editorial by Elder [55]. As much evidence becomes available to show that we are not influencing the outcome of VUR, less VCUGs may be ordered [7, 93]. Possible alternatives include Doppler ultrasound [94] or ultrasound with contrast [61]. A suprapubic approach to avoid catheterisation seems promising but still requires filling and voiding [49]. Methods to detect reflux without voiding are impaired as some reflux may be present only on voiding [95], although the fact that whether this is important or not is debated [96]. Nuclear medicine scanning may be an alternative or may be able to select those who are more likely to benefit from VCUG [67]. Nuclear medicine cystoscopy replaces radio-opaque contrast with pharmacolabelled material with lower radiation, but otherwise it is very similar to the VCUG. Currently, VCUG remains the gold standard until less invasive tests are developed [1]. At the very least, we should be perfecting our current technique [12].

## 5. DISTRESS, PAIN, AND ANTICIPATORY ANXIETY

**Figure 1:**
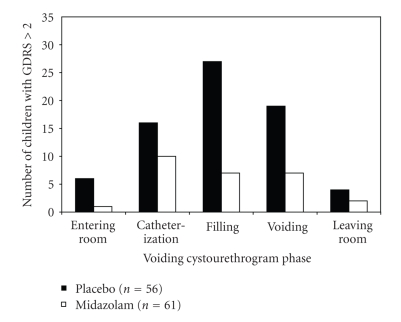
Bar graph shows the number of children (*n* = 117) who experienced serious or severe distress (Groningen distress rating scale (GDRS) score >2 [90]) at each phase of voiding cystourethrography. 56 received placebo (black bars) and 61 received midazolam (white bars), from [45].

Distress is an all encompassing term that may or may not include a painful stimulus. This can be evidenced by fear of a procedure, pain responses prior to nociceptive stimuli, or anxiety behaviours before a planned event. Distress during the voiding cystourethrogram has been reported in observational and controlled studies. Phillips et al. [13] showed that 52 out of 73 children (71%) experienced serious distress, severe distress, or panic on the Groningen distress rating scale [90]. Herd et al. found that serious or severe distress was detected in 61% of all unsedated children at some stages during the VCUG. This level of distress may have been brief but is generally considered unacceptable. This distress is caused not only by urethral catheterisation, but also by the distension of the bladder and the subsequent voiding of urine in a socially abnormal situation (Figure 1). Nociceptors related to urethral mucosa and stretch receptors in the bladder provide the peripheral pain signals, but the majority of the distress is cortical.

Distress can also be manifest in the parent. Parents' perceptions of fear, distress, and pain in their children are anticipated to be greater than the reality [51].

## 6. PSYCHOLOGICAL THERAPY (NONPHARMACOLOGICAL TREATMENTS)

Psychological treatments should be considered first as they often have little or no risk. There are many ways to reduce the distress of procedures in children [19] and even more challenges researching and implementing psychological interventions for controlling children's pain [97]. Interventions may range between simple educational [28] and more structured play therapy sessions**[14, 76] and hypnoses [42]. Preparation, distraction, and reassurance before, during, and after the procedure are likely to reduce the distress of the procedure [22, 69, 75]. Parental presence is comforting for children during painful procedure and should be encouraged despite the lack of specific VCUG evidence [23].

Those who have been previously distressed by VCUG would seem to be ideal candidates for sedation, but the majority of children would not have had a previous VCUG. Factors which may reduce distress in children during VCUG include “effortful control” by the child and coping and distress-promoting behaviours by the parent [34]. No validated prediction tool exists for VCUG distress.

## 7. PHARMACOTHERAPY

Pharmacotherapy includes sedation, anxiolysis, analgesia, amnesia, and anesthesia.

### 7.1. Sedating agents

Sedation continues to be difficult to define[63]. In the case of the VCUG, a degree of consciousness is required. This may be defined as light sedation, and the use of oxymoron “conscious sedation” is being discouraged [98].

**Midazolam**

Of the selected studies, 5 had midazolam as a treatment arm and 4 of which were oral and one intranasal. Oral midazolam dose was 0.5 mg/kg in two studies [45, 47] and 0.6 mg/kg in two other studies [41, 43]. Maximum dose was 15 mg in all. Time between ingestion and procedure ranged from 15 to 30 minutes. Intranasal dose in one study was 0.2 mg/kg with a maximum of 5 mg, and it was administered 5 minutes before the procedure [52]. A number of behavioural measures were employed (Table 1). All the studies demonstrate significantly less distress with midazolam in a variety of measurement tools. Few adverse effects were encountered. Midazolam may cause adverse paradoxical agitated reactions in less than 5% of children [99]. These reactions have been shown in case reports to be ameliorated using the antidote for midazolam (flumazenil) both in adults [100] and children [101]. Ketamine, a dissociative anesthetic, has been shown to be more effective than increased doses of midazolam or placebo in a randomised controlled trial [99].

The study by Stockland et al. [52] on 95 children compared intranasal midazolam (0.2 mg/kg with a maximum of 5 mg) to placebo. Nurses reported a trend to easier procedure in the midazolam group (*P* = .07), with girls reported easier than boys (*P* = .06). No serious adverse events were reported. Parents felt that the administration of midazolam was more uncomfortable than that of placebo (*P* < .001). Parents felt that midazolam made catheterisation, voiding, and the overall procedure more comfortable (*P* = .015, *P* = .08, and *P* = .047, resp.). The authors report *P*-values and no absolute scores, which makes it impossible to estimate treatment effect size or clinical relevance.

A study by the current author and colleagues [45] compared oral midazolam (0.5 mg/kg with a maximum of 15 mg) to placebo in 125 children who had VCUG. Behavioural observations were completed in 117. This was the only study that calculated a priori power requirement or attempted to quantify the treatment effect. We rated our paper highly using the Jadad score [86]. We found no serious adverse events. The number of children experiencing serious or severe distress (Groningen distress rating scale (GRDS) >2) at any stage of the procedure was 34 (61%) in the placebo group and 16 (26%) in the midazolam group. Number needed to treat to reduce serious or severe distress in one child was 2.9 (95%CI 1.9–5.5). VUR was identified in 16% of all children. This study was limited to children above the age of one year.

**Nitrous oxide (N_2_O)**

Two studies evaluated nitrous oxide given with continuous flow devices at 50% and 70%. Keidan et al. compared 50% nitrous oxide in 23 children to 0.5 mg/kg oral midazolam in 24 children without a placebo group [47]. They found no difference between midazolam and 50% nitrous oxide although they did not design this as an equivalence study, and no power calculation was done. There was a trend for the time to micturition to be longer in the nitrous group (15.3 minutes versus 7.2 minutes), but it did not reach statistical significance (*P* = .08). Nitrous oxide was significantly faster with regard to recovery time, with recovery in 29 minutes versus 63 minutes (*P* < .001). Zier et al. used 70% continuous nitrous oxide only for urethral catheterisation phase of VCUG in an older group of 107 children, and compared this to standard treatment in other 107 children [59]. The authors chose not to randomise the study based on difficulties with recruitment and parental expectations. Brief behavioural distress scores (BBDSs) were demonstrated by the observational tool selected [102]. Wong-Baker FACES scale was the self-report tool used [103]. For the VCUG group (*n* = 101), BBDS was 44 (range of 11–100) in the nonsedated group versus 11 (range of 0–67) for the sedated group (*P* < .001). Immediately after catheterisation, the Wong-Baker FACES scale median was 6 for the nonsedated group and 0 for the sedated group (*P* < .001). Both studies reported time of completion, but neither study reported VUR grading or residual volume.

**Other agents**

Choral hydrate was compared to oral midazolam and placebo in one study [41]. A dose of 25 mg/kg was not found to be statistically different from placebo in reducing distress. This may have been due to inadequate dose or lack of power in the study. The sedation scale was also not significant for chloral versus placebo and, therefore, it suggests too low a dose was selected. There is no enough data to make any assessment of effect on voiding dynamics.

One retrospective study of propofol using historical controls was selected for review [48]. While this was an attempt to create a sedative state using low-dose propofol, the study required the presence of an anesthetist. During this study, low-dose propofol infusion followed sevoflurane gas induction and intravenous cannula insertion. This study found that propofol reduced the ability of children to completely void, which may interfere with the diagnosis of VUR.

### 7.2. Anxiolysis

Midazolam in the doses used in the reviewed studies is anxiolytic. Many children may appear fully conscious yet more cooperative, while another child given the same dose may appear sleepy. Where anxiolysis ends and sedation begins is unclear, but there would be a large overlap.

### 7.3. Analgesia

There is a wide range of analgesics available for children [104]. Midazolam does not provide any analgesia and, therefore, should be supplemented with a simple analgesic.

**Acetaminophen**

Acetaminophen is the most commonly provided childhood analgesic with low side effects and cost. It is routinely offered prior to other potentially painful procedures in children such as vaccination. Acetaminophen is usually provided in a sweet syrup base, and could be used to disguise the bitter taste of midazolam. There are many formulations of acetaminophen syrup, and palatability may vary [105].

**Oral sucrose**

Oral sucrose is an effective analgesic in new-born babies, and has been subject to several controlled trials and a Cochrane review [106]. While no studies have examined its effect for VCUG distress, it seems a simple likely effective intervention with low risk for children under 3 months of age.

**Nitrous oxide**

Nitrous oxide is a strong analgesic antagonising central NMDA receptors, and this is a potential advantage over midazolam.** Study of Keidan et al. comparing midazolam to continuous flow nitrous oxide found no difference in FLACC scores [91], a measure of pain used more recently for procedural distress [107]. Study of Keidan et al. was not designed as an equivalence study, and no power calculations were done; so a true difference may not have been detected by the study.

**Opiates**

No studies have looked at opiate use for VCUG distress. Intranasal midazolam has proven effective, and opiates may also be administered by this route. Intranasal fentanyl shows promise as a rapid, easy-to-administer analgesic for severe pain in the children's Emergency Department [108]. Opiates may interfere with bladder function [109].

**Local anesthetics**

Lignocaine gel has been shown to reduce the pain of catheterisation for VCUG, but a 10-minute process of repeated application of lignocaine gel to the urethral meatus is required. The authors did not measure the effect of this procedure but only the reduced pain of catheterisation that followed. It would seem reasonable to use it with low risk of harm but at added cost [66]. Further study on children is required.

### 7.4. Anesthesia

There is increasing use of deeper sedation outside the operating room by nonanesthesiologists [110]. There is debate about which agents should be used outside the operating room and who should provide this service [63]. For VCUG, anesthetics have been given to avoid the trauma associated with urethral catheterisation, and then the child is allowed to wake and complete the VCUG. This does not avoid the distress caused by bladder distension or micturition. It also requires an anesthetist and the full costs associated with anesthesia and recovery.

## 8. WHO SHOULD RECEIVE SEDATION?

Many children do not experience distress during the VCUG. This may be related to previous experience, coping style, parental influence, staff skill, and empathy. Developmental considerations and education level of the child and parent are important. Nevertheless, many children, who would not have been predicted, may go on to experience distress. Parental perceptions of the procedure are such that most parents would request some medication if it were effective, safe, and available [43, 47].

## 9. DOES SEDATION AFFECT THE ABILITY OF THE VCU TO DIAGNOSE REFLUX?

Effect of sedation on ability to void can be measured with indirect or direct measures. Indirect measures include filling volume, residual volume, and time of micturition. Bozkurt et al. carefully examined urodynamic variables under the influence of midazolam [62]. They used a high-intranasal dose of 0.5 mg/kg. Stockland et al. used intranasal midazolam at a dose of 0.2 mg/kg, and found no difference in reflux grading between the groups [52]. They did not perform a power calculation, so there is still the possibility of missing a true effect. Herd et al. considered a clinically important difference in VUR to be a true shift of one grade down by half of the subjects with the use of midazolam [45]. It was important to detect a difference, so a 90% power was used. There was no difference in VUR grading between the groups (nonlinear mixed model analysis, *P* = .31). There was no evidence of a difference in volume infused between the two groups (*P* = .08).

## 10. CONCLUSIONS

Sedation reduces distress of the micturating cystourethrogram in children previously distressed or likely to be distressed. Midazolam is the agent most studied, and has an excellent safety profile. An oral dose of 0.5-0.6 mg/kg or intranasal dose of 0.2 mg/kg seems effective. Most children have not had a VCUG previously, and it may be difficult to predict which of them will go on to have distress. When giving oral midazolam of 0.5 mg/kg to children routinely, the number needed to treat them is 2.9 (95%CI 1.9–5.5) to eliminate serious or severe distress. Continuous flow nitrous oxide appears promising, particularly with a fast onset and recovery time, but it has greater potential for deeper sedation. This may interfere with voiding, and further studies are required. Midazolam appears not to interfere with the VCUG's ability to diagnose vesicoureteric reflux using indirect (residual volume) and direct (VUR grading) measures. There are many children who would avoid distress if they were given sedation. Local sedation services should be engaged, and safety guidelines should be followed to ensure that this effective treatment might be implemented safely.

## Figures and Tables

**Table 1:** Studies comparing a sedative against another sedative, placebo, or standard treatment for VCUG. Design and interventions. (NSD: no significant difference; VCUG: voiding cystourethrogram; RNC: radionuclide cystography; VUR: vesicoureteric reflux; SD: standard deviation).

Authors; year; country	Title	Patients	Intervention and comparison	Nonpharmacological cointervention	Time of follow-up and differences (if any)
I. Akil, M. Ozkol,O. Y. Ikizoglu, M. Polat, O. Y. Tuncyurek, O. Taskin, H. Yuksel; 2005; Turkey [41]	“Premedication during micturating cystourethrogram to achieve sedation and anxiolysis”	53 (39F;14M), >6 m, median age of 6 y (range from 7 m to 11.1 y); first VCUG 98%	Oral *midazolam* of 0.6 mg/kg (max. of 15 mg) versus *chloral hydrate* of 25 mg/kg (max. of 500 mg) versus *Placebo*; 15–30 min prior to VCUG	Parents informed about MCUG and permission granted for sedative drug and making child nil by mouth for 3 h, parental presence not stated	Until they were allowed to drink clear liquids, usually 1 h after completion of the procedure

J. S. Elder, R. Longenecker; 1995; USA [43]	“Premedication with oral midazolam for voiding cystourethrography in children: safety and efficacy”	98 children previously distressed by VCUG (38) or appeared shy (79F;19M), mean age of 4.4 (range from 23 m to 9 y); 25 controls (21F : 4M), mean age of 4.6 (range not stated); first VCUG 61%	Oral *midazolam* of 0.6 mg/kg (max. of 15 mg), 20–30 min prior to VCUG or NUC versus *standard care*	Parents of intervention group-contacted prior with purpose of midazolam and expected effects, parents are allowed to be present	Phone call at 48 h

D. W. Herd, K. A. McAnulty, N. A. Keene, D. E. Sommerville; 2006; New Zealand [45]	“Conscious sedation reduces distress in children undergoing voiding cystourethrography and does not interfere with the diagnosis of vesicoureteric reflux: a randomized controlled study”	Children of 1–14 y (eligible); 139 randomised, 117 completed VCUG on the day (84F : 33M), 8 had VCUG completed later, age >1 y, mean ages of 3.6 y (SD1.8) and 3.4 y (SD2.1), ASAI-II	Oral *midazolam* of 0.5 mg/kg (max. of 15 mg), 30 min prior to catheter insertion versus *placebo*	All offered play therapy (visit to department, doll catheterised), four-page pamphlet, only the treatment group fasted for 6 h with solids and 4 h with liquid (i.e., control group was allowed to eat), parent/caregiver present, skilled nurse did all catheters	60–90 min after medication

I. Keidan, R. Zaslansky, M. Weinberg, A. Ben-Shlush, J. M. Jacobson, A. Augarten, Y. Mor; 2005; Israel [47]	“Sedation during voiding cystourethrography: comparison of the efficacy and safety of using oral midazolam and continuous flow nitrous oxide”	47 (42F : 5M), age of 3–16 y, ASAI and II, mean age of 6 (range from 3 to 15)	Oral *midazolam* of 0.5 mg/kg (max. of 15 mg), 20 min prior to procedure versus** continuous flow *50*% *nitrous oxide*	Both groups fasted with solids for 6 h, and liquids for 2 h, parents were encouraged to accompany the children throughout the procedure, flavoured nasal mask was used for nitrous oxide	24 h follow-up by telephone, recovery time of 63 min (SD 25) in midazolam group, 29 min (SD 10) in the N2O group (*p* < .001)

P. A. Merguerian, S. T. Corbett, J. Cravero; 2006; USA [48]	“Voiding ability using propofol sedation in children undergoing voiding cystourethrograms: a retrospective analysis”	544 charts, 287 selected ages from 2 to 8 (preselected), mean age of 51 m (244F : 43M), first VCUG 75%	Sevoflurane induction followed by *propofol* infusion on its own (*historical controls*)	Not reported	

E. Stokland, S. Andréasson, B. Jacobsson, U. Jodal, B. Ljung; 2003; Sweden [52]	“Sedation with midazolam for voiding cystourethrography in children: a randomised double-blind study”	Children of 0.5 to 9 y (eligible), 95 enrolled (70F : 20M), gender stratified, median age of 2.2 y, midazolam, 3.2 placebo	Intranasal *midazolam* of 0.2 mg/kg (max. of 5 mg), 3–5 min prior to bladder catheter versus *placebo*	Oral and written information	Follow-up questionnaire and phone call at 48 h

J. L. Zier, K. A. Kvam, S. C. Kurachek, M. Finkelstein; 2007; USA [59]	“Sedation with nitrous oxide compared with no sedation during catheterization for urologic imaging in children”	Children of 4–18 y selected by investigator undergoing VCUG or RNC, enrolled 204 (165F : 39M) out of 389, mean age nonsedated: 6.4 (range of 4–15.2), sedated: 6.3 (range of 4–14.9)	Continuous flow *70*% *nitrous oxide* until catheterisation is complete versus *standard care*	All patients fasted for 4 h	To time of discharge, longer in sedated group, 85 min versus 33 min (*P* < 0.001)

**Table 2:** Studies comparing a sedative against another sedative, placebo, or standard treatment for VCUG. Outcomes, results, and follow-up. (NSD: no significant difference; VCUG: voiding cystourethrogram; RNC: radionuclide cystography; VUR: vesicoureteric reflux; SD: standard deviation).

Authors; year; country	Sedation score outcome and results	Distress outcome(s) and results	Urological outcome(s) and results	Safety outcome(s) and adverse events
I. Akil, M. Ozkol, O. Y. Ikizoglu, M. Polat, O. Y. Tuncyurek, O. Taskin, H. Yuksel; 2005; Turkey [41]	Breitkopf and Buttner classification of emotional status [87], 1.87 (SD0.72) in midazolam versus 1.35 (SD0.49) in control (*P* = .01), duration of sedation is 68 min (midazolam), 28 min (chloral), *P* < .001	Frankl behaviour rating score [88] NSD; Spielberger's state anxiety inventory [41] NSD; Houpt behaviour scale [88] of 4.93 (SD1.12) in midazolam group versus control of 4.12 (SD 1.05) in chloral group, all NSD	Postvoid residual volume, VCUG grading, no difference found	None found, defined as drop in PaO2/Sats by 5%, systolic blood pressure drop of 15 mm Hg, drop in pulse to 60 bpm

J. S. Elder, R. Longenecker; 1995; USA [43]	None	Phone call at 48 h, recall, behavioural side effects; parental wishes, 97 out of 98 contacted, 56 children (60%), no recall of VCUG, 19 (21%) recalled parts, 10 remembered the procedure without negative experience, 9 recalled a negative experience, 12 out of 97 children had behavioural side effects reported after the study, 92 out of 97 (95%) parents of sedated children would request the use of midazolam again	Postvoid residual volume (Bis and Slovis method [89]), no residual volume in 74% of midazolam group and 72% of control group; NSD	Saturation decrease by 10%, systolic BP drop by 15 mm Hg, respective rate down to 8/min, HR down to 60/min, one child had a transient decrease in saturation requiring no intervention

D. W. Herd, K. A. McAnulty, N. A. Keene, D. E. Sommerville; 2006; New Zealand [45]	None	Independent observer Groningen distress rating scale [90]; nursing GDRS; heart rate; parent-child interaction, 61% of placebo group experienced serious or severe distress (GDRS of 3 or 4); 26% of midazolam group had the same distress; number needed for treatment was 2.9 (95%CI 1.9–5.5)	VUR grade; volume infused, no difference in volume infused (*P* = .8), no difference in VUR grading (*P* = .31), a priori power of 90%	Oxygen requirement (Sats <94%), two children in midazolam group had transient desaturations to less than 94% and were given oxygen

I. Keidan, R. Zaslansky, M. Weinberg, A. Ben-Shlush, J. M. Jacobson, A. Augarten, Y. Mor; 2005; Israel [47]	AVPU (alert, responds to voice, responds to pain, unresponsive)	FLACC (face, legs, activity, crying, consolability) score for pain [91]; anxiety score (observer scale of behavioural distress) [92]; no difference between midazolam and nitrous oxide, number of children requiring physical restraint is 10/24 in midazolam and 2/23 for N2O (*P* = .01)	Time to micturition 7.2 (SD2.5) min for midazolam and 15.3 (SD 17.3), *P* = .8	Oxygen saturation <93%, alteration in heart rate or BP by 15% from baseline, oversedation defined as “U” on the AVPU scale

P. A. Merguerian, S. T. Corbett, J. Cravero; 2006; USA [48]	Not reported	None	Void to completion; sedated children (55%) could void to completion compared to 89% nonsedated (*P* < .001)	Not reported

E. Stokland, S. Andréasson, B. Jacobsson, U. Jodal, B. Ljung; 2003; Sweden [52]	None	VAS from 0 mm to 100 mm (severe problems); nurse observation VAS NSD, parent VAS, administration of midazolam more uncomfortable (*P* < .001), catheter, and overall procedure more uncomfortable with placebo (*P* < .001); parent follow-up questionnaire at 12, 24, and 48 h of “reactions,” NSD	VUR grade; volume infused; ability to void; NSD	Not defined, none reported

J. L. Zier, K. A. Kvam, S. C. Kurachek, M. Finkelstein; 2007; USA [59]	None	Brief behavioural distress score (BBDS) for VCUG, median age of 44 (range of 11–100) nonsedated, 11 (range of 0–67) sedated (*P* < .001), patient self-reported Wong-Baker FACES pain rating scale, 6 after catheter in nonsedated, 0 in sedated (*P* < .001)	Time to bladder emptying; NSD	One patient in sedated group experienced nausea, no desaturations

**Table 3:** Quality assessment of studies of sedation for VCUG, including Jadad score [86].

Authors; year; country; reference	Randomised	Randomisation described	Blinded	Allocation concealment	Withdrawals and dropouts?	Control	Placebo group	A priori power	Jadad score [86]
I. Akil, M. Ozkol, O. Y. Ikizoglu, M. Polat, O. Y. Tuncyurek, O. Taskin, H. Yuksel; 2005; Turkey [41]	Quasi	Y	Y (not described as double blind)	Cherry-flavoured liquid	N	Y	Y	N	1 (poor)

J. S. Elder, R. Longenecker; 1995; USA [43]	N	—	N	Kool-aid (artificial sweetner)	N	Y	Y	N	0 (poor)

D. W. Herd, K. A. McAnulty, N. A. Keene, D. E. Sommerville; 2006; New Zealand [45]	Y	Y	Y (blinding analysis done)	Mango and orange juices	Y	Y	Y	Y; 80% for GDRS; 90% for VUR grade	5 (excellent)*

I. Keidan, R. Zaslansky, M. Weinberg, A. Ben-Shlush, J. M. Jacobson, A. Augarten, Y. Mor; 2005; Israel [47]	Y	N	N	—	N	Y	N	N	1 (poor)

P. A. Merguerian, S. T. Corbett, J. Cravero; 2006; USA [48]	N	—	—	—	—	Y (historical)	N	N	0 (poor)

E. Stokland, S. Andréasson, B. Jacobsson, U. Jodal, B. Ljung; 2003; Sweden [52]	Y	Y	Y	Described	N	Y	Y	N	4 (good)

J. L. Zier, K. A. Kvam, S. C. Kurachek, M. Finkelstein; 2007; USA [59]	N	—	N	—	Y	Y	Y	N	1 (poor)

*Authors' self-score.

## References

[1] Bergman DA, Baltz RD, Cooley JR (1999). Practice parameter: the diagnosis, treatment, and evaluation of the initial urinary tract infection in febrile infants and young children. *Pediatrics*.

[2] Bundy DG (2007). Vesicoureteral reflux. *Pediatrics in Review*.

[3] Elder JS, Snyder HM, Peters C (1992). Variations in practice among urologists and nephrologists treating children with vesicoureteral reflux. *The Journal of Urology*.

[4] Craig JC, Irwig LM, Knight JF, Roy LP (2000). Does treatment of vesicoureteric reflux in childhood prevent end-stage renal disease attributable to reflux nephropathy?. *Pediatrics*.

[5] Faust WC, Pohl HG (2007). Role of prophylaxis in vesicoureteral reflux. *Current Opinion in Urology*.

[6] Garin EH, Young L (2007). Much pain, little gain from voiding cystourethrogram after urinary tract infection. *Pediatrics*.

[7] Hodson EM, Wheeler DM, Vimalchandra D, Smith GH, Craig JC (2007). Interventions for primary vesicoureteric reflux. *Cochrane Database of Systematic Reviews*.

[8] Newman TB (2006). Much pain, little gain from voiding cystourethrograms after urinary tract infection. *Pediatrics*.

[9] Jones KV (2005). Time to review the value of imaging after urinary tract infection in infants. *Archives of Disease in Childhood*.

[10] Wald ER (2006). Vesicoureteral reflux: the role of antibiotic prophylaxis. *Pediatrics*.

[11] Wald ER (2006). Much pain, little gain from voiding cystourethrograms after urinary tract infection: in reply. *Pediatrics*.

[12] Agrawalla S, Pearce R, Goodman TR (2004). How to perform the perfect voiding cystourethrogram. *Pediatric Radiology*.

[13] Phillips D, Watson AR, Collier J (1996). Distress and radiological investigations of the urinary tract in children. *European Journal of Pediatrics*.

[14] Phillips DA, Watson AR, MacKinlay D (1998). Distress and the micturating cystourethrogram: does preparation help?. *Acta Paediatrica*.

[15] Stashinko EE, Goldberger J (1998). Test or trauma? The voiding cystourethrogram experience of young children. *Issues in Comprehensive Pediatric Nursing*.

[16] Hjelm-Karlsson K (1991). Dispelling the fear of the unknown. Effects of information to patients undergoing urography. *Acta Radiologica, Supplement*.

[17] Jay SM, Elliott CH (1990). A stress inoculation program for parents whose children are undergoing painful medical procedures. *Journal of Consulting and Clinical Psychology*.

[18] Jay SM, Elliott CH, Ozolins M, Olson RA, Pruitt SD (1985). Behavioral management of children's distress during painful medical procedures. *Behaviour Research and Therapy*.

[19] Kuttner L (1989). Management of young children's acute pain and anxiety during invasive medical procedures. *Pediatrician*.

[20] Lang EV, Benotsch EG, Fick LJ (2000). Adjunctive non-pharmacological analgesia for invasive medical procedures: a randomised trial. *The Lancet*.

[21] Lang EV, Joyce JS, Spiegel D, Hamilton D, Lee KK (1996). Self-hypnotic relaxation during interventional radiological procedures: effects on pain perception and intravenous drug use. *International Journal of Clinical and Experimental Hypnosis*.

[22] Manne SL, Redd WH, Jacobsen PB, Gorfinkle K, Schorr O, Rapkin B (1990). Behavioral intervention to reduce child and parent distress during venipuncture. *Journal of Consulting and Clinical Psychology*.

[23] Piira T, Sugiura T, Champion GD, Donnelly N, Cole ASJ (2005). The role of parental presence in the context of children's medical procedures: a systematic review. *Child: Care, Health and Development*.

[24] Pretzlik U, Sylva K (1999). Paediatric patients' distress and coping: an observational measure. *Archives of Disease in Childhood*.

[25] Pretzlik U, Sylva K (1999). Paediatric patients' distress and coping during medical treatment: a self report measure. *Archives of Disease in Childhood*.

[26] Stephens BK, Barkey ME, Hall HR (1999). Techniques to comfort children during stressful procedures. *Accident and Emergency Nursing*.

[27] Zelikovsky N, Rodrigue JR, Gidycz CA (2001). Reducing parent distress and increasing parent coping-promoting behavior during children's medical procedure. *Journal of Clinical Psychology in Medical Settings*.

[28] Zelikovsky N, Rodrigue JR, Gidycz CA, Davis MA (2000). Cognitive behavioral and behavioral interventions help young children cope during a voiding cystourethrogram. *Journal of Pediatric Psychology*.

[29] Kauffman RE, Banner W, Berlin CM (1992). Guidelines for monitoring and management of pediatric patients during and after sedation for diagnostic and therapeutic procedures. *Pediatrics*.

[30] Gross JB, Bailey PL, Caplan RA (1996). Practice guidelines for sedation and analgesia by non-anesthesiologists: a report by the American Society of Anesthesiologists Task Force on sedation and analgesia by non-anesthesiologists. *Anesthesiology*.

[31] Gorman R, Bates BA, Benitz WE (2002). Guidelines for monitoring and management of pediatric patients during and after 
sedation for diagnostic and therapeutic procedures: addendum. *Pediatrics*.

[32] Coté CJ, Karl HW, Notterman DA, Weinberg JA, McCloskey C (2000). Adverse sedation events in pediatrics: analysis of medications used for sedation. *Pediatrics*.

[33] Coté CJ, Notterman DA, Karl HW, Weinberg JA, McCloskey C (2000). Adverse sedation events in pediatrics: a critical incident analysis of contributing factors. *Pediatrics*.

[34] Salmon K, Pereira JK (2002). Predicting children's response to an invasive medical investigation: the influence of effortful control and parent behavior. *Journal of Pediatric Psychology*.

[35] Boswinkel JP, Litman RS (2005). Sedating patients for radiologic studies. *Pediatric Annals*.

[36] Krauss B, Green SM (2000). Sedation and analgesia for procedures in children. *The New England Journal of Medicine*.

[37] Krauss B, Green SM (2006). Procedural sedation and analgesia in children. *The Lancet*.

[38] Safe Sedation of Children Undergoing Diagnostic and Therapeutic Procedures: A national clinical guideline (SIGN 58). http://www.sign.ac.uk/guidelines/fulltext/58/index.html.

[39] Guidelines on Sedation and/or Analgesia for Diagnostic and Interventional Medical or Surgical Procedures. Australia and New Zealand College of Anaesthetists. 
Available at. http://www.anzca.edu.au/resources/professional-documents/professional-standards/ps9.html.

[40] Coté CJ, Wilson S, Casamassimo P (2006). Guidelines for monitoring and management of pediatric patients during and after 
sedation for diagnostic and therapeutic procedures: an update. *Pediatrics*.

[41] Akil I, Ozkol M, Ikizoglu OY (2005). Premedication during micturating cystourethrogram to achieve sedation and anxiolysis. *Pediatric Nephrology*.

[42] Butler LD, Symons BK, Henderson SL, Shortliffe LD, Spiegel D (2005). Hypnosis reduces distress and duration of an invasive medical procedure for children. *Pediatrics*.

[43] Elder JS, Longenecker R (1995). Premedication with oral midazolam for voiding cystourethrography in children: safety and efficacy. *American Journal of Roentgenology*.

[44] Ellerkmann RM, Dunn JS, McBride AW (2003). A comparison of anticipated pain before and pain rating after the procedure in patients who undergo cystourethroscopy. *American Journal of Obstetrics and Gynecology*.

[45] Herd DW, McAnulty KA, Keene NA, Sommerville DE (2006). Conscious sedation reduces distress in children undergoing voiding cystourethrography and does not interfere with the diagnosis of vesicouteric reflux: a randomized controlled study. *American Journal of Roentgenology*.

[46] Kadioglu A (2004). Voiding cystourethrography: sedation or no sedation?. *Pediatric Radiology*.

[47] Keidan I, Zaslansky R, Weinberg M (2005). Sedation during voiding cystourethrography: comparison of the efficacy and safety of using oral midazolam and continuous flow nitrous oxide. *The Journal of Urology*.

[48] Merguerian PA, Corbett ST, Cravero J (2006). Voiding ability using propofol sedation in children undergoing voiding cystourethrograms: a retrospective analysis. *The Journal of Urology*.

[49] Oswald J, Riccabona M, Lusuardi L, Ulmer H, Bartsch G, Radmayr C (2002). Voiding cystourethrography using the suprapubic versus transurethral route in infants and children: results of a prospective pain scale oriented study. *The Journal of Urology*.

[50] Robinson M, Savage J, Stewart M, Sweeney L (1999). The diagnostic value, parental and patient acceptability of micturating cysto-urethrography in children. *Irish Medical Journal*.

[51] Srivastava T, Betts G, Rosenberg AR, Kainer G (2001). Perception of fear, distress and pain by parents of children undergoing a micturating cystourethrogram: a prospective study. *Journal of Paediatrics and Child Health*.

[52] Stokland E, Andréasson S, Jacobsson B, Jodal U, Ljung B (2003). Sedation with midazolam for voiding cystourethrography in children: a randomised double-blind study. *Pediatric Radiology*.

[53] Bjørkholen EC, Gravdahl CØ, Vandvik IH (2005). Micturating cystourethrography: are the practical routines in accordance with empirical knowledge?. *Tidsskrift for den Norske Laegeforening*.

[54] Chen E (2006). Commentary: the role of memory in managing children's distress during medical procedures. *Journal of Pediatric Psychology*.

[55] Elder JS (2005). Imaging for vesicoureteral reflux—is there a better way?. *The Journal of Urology*.

[56] Madzik J, Marciński A, Brzewski M (2006). Midazolam administration at a department of pediatric radiology: conscious sedation for diagnostic imaging studies. *Polish Journal of Radiology*.

[57] Radmayr C (2005). Can hypnosis reduce distress and improve compliance with voiding cystourethrogram in children?. *Nature Clinical Practice Urology*.

[58] Schmit P, Sfez M (1997). Pain and stress in pediatric uroradiology: efficacy of a specific protocol. *Journal de Radiologie*.

[59] Zier JL, Kvam KA, Kurachek SC, Finkelstein M (2007). Sedation with nitrous oxide compared with no sedation during catheterization for urologic imaging in children. *Pediatric Radiology*.

[60] (2006). Guideline statement: management of procedure-related pain in children and adolescents. *Journal of the Paediatrics and Child Health*.

[61] Bosio M (1998). Cystosonography with echocontrast: a new imaging modality to detect vesicoureteric reflux in children. *Pediatric Radiology*.

[62] Bozkurt P, Kiliç N, Kaya G, Yeker Y, Eliçevik M, Söylet Y (1996). The effects of intranasal midazolam on urodynamic studies in children. *British Journal of Urology*.

[63] Coté CJ (2008). Round and round we go: sedation—what is it, who does it, and have we made things safer for children?. *Paediatric Anaesthesia*.

[64] Diament MJ, Stanley P (1987). The use of midazolam for sedation of infants and children. *American Journal of Roentgenology*.

[65] Garin EH, Olavarria F, Nieto VG, Valenciano B, Campos A, Young L (2006). Clinical significance of primary vesicoureteral reflux and urinary antibiotic prophylaxis after acute pyelonephritis: a multicenter, randomized, controlled study. *Pediatrics*.

[66] Gerard LL, Cooper CS, Duethman KS, Gordley BM, Kleiber CM (2003). Effectiveness of lidocaine lubricant for discomfort during pediatric urethral catheterization. *The Journal of Urology*.

[67] Hansson S, Dhamey M, Sigström O (2004). Dimercapto-succinic acid scintigraphy instead of voiding cystourethrography for infants with urinary tract infection. *The Journal of Urology*.

[68] Ilan K, Zaslansky R, Weinberg M (October 2004). Sedation during voiding cystourethrography: comparison of the efficacy and safety of using oral midazolam and continuous-flow nitrous oxide. *Section on Urology*.

[69] Kleiber C, McCarthy AM (1999). Parent behavior and child distress during urethral catheterization. *Journal of the Society of Pediatric Nurses*.

[70] Ljung B, Andréasson S (1996). Comparison of midazolam nasal spray to nasal drops for the sedation of children. *Journal of Nuclear Medicine Technology*.

[71] Ljungman G, Kreuger A, Andréasson S, Gordh T, Sörensen S (2000). Midazolam nasal spray reduces procedural anxiety in children. *Pediatrics*.

[72] Merritt KA, Ornstein PA, Spicker B (1994). Children's memory for a salient medical procedure: implications for testimony. *Pediatrics*.

[73] Quas JA, Goodman GS, Bidrose S, Pipe M-E, Craw S, Ablin DS (1999). Emotion and memory: children's long-term remembering, forgetting, and suggestibility. *Journal of Experimental Child Psychology*.

[74] Rubenstein JN, Maizels M, Kim SC, Houston JTB (2003). The PIC cystogram: a novel approach to identify “occult” vesicoureteral reflux in children with febrile urinary tract infections. *The Journal of Urology*.

[75] Salmon K, McGuigan F, Pereira JK (2006). Brief report: Optimizing children's memory and management of an invasive medical procedure: the influence of procedural narration and distraction. *Journal of Pediatric Psychology*.

[76] Salmon K, Price M, Pereira JK (2002). Factors associated with young children's long-term recall of an invasive medical procedure: a preliminary investigation. *Journal of Developmental and Behavioral Pediatrics*.

[77] Schmit P, Sfez M (1997). Management of anxious and painful manifestations in pediatric uroradiology. *Journal of Radiology*.

[78] Smellie J, Edwards D, Hunter N, Normand ICS, Prescod N (1975). Vesico ureteric reflux and renal scarring. *Kidney International*.

[79] Stein M, Lubetkin D, Taub HC, Skinner WK, Haberman J, Kreutzer ER (1994). The effects of intraurethral lidocaine anesthetic and patient anxiety on pain perception during cystoscopy. *The Journal of Urology*.

[80] Stokland E, Andréasson S, Jacobsson B, Jodal U, Ljung B (2004). Voiding cystourethrography: sedation or no sedation?: in reply. *Pediatric Radiology*.

[81] Thompson M, Simon SD, Sharma V, Alon US (2005). Timing of follow-up voiding cystourethrogram in children with primary vesicoureteral reflux: development and application of a clinical algorithm. *Pediatrics*.

[82] Sobczak OM (1972). General anesthesia in outpatient pediatric uroradiology. *Anesthesia & Analgesia*.

[83] Webb E, Goodwin WE (1973). Anesthesia for voiding cystourethrograms in pediatric patients. *The Journal of Urology*.

[84] Weiss H, Badlani G (1993). Effects of anesthesia on micturition and urodynamics. *International Anesthesiology Clinics*.

[85] Woodard JR, Filardi G (1976). The demonstration of vesicoureteral reflux under general anesthesia. *The Journal of Urology*.

[86] Jadad AR, Moore RA, Carroll D (1996). Assessing the quality of reports of randomized clinical trials: is blinding necessary?. *Controlled Clinical Trials*.

[87] Cooper J, Jobling D, Edmunds DH (1978). Sedation for minor oral surgery: inhalation sedation with 25 per cent nitrous oxide. *Journal of Dentistry*.

[88] Hosey MT, Blinkhorn AS (1995). An evaluation of four methods of assessing the behaviour of anxious child dental patients. *International Journal of Paediatric Dentistry*.

[89] Bis KG, Slovis TL (1990). Accuracy of ultrasonic bladder volume measurement in children. *Pediatric Radiology*.

[90] Humphrey GB, Boon CMJ, van Linden van den Heuvell GF, van de Wiel HBM (1992). The occurrence of high levels of acute behavioral distress in children and adolescents undergoing routine venipunctures. *Pediatrics*.

[91] Merkel SI, Voepel-Lewis T, Shayevitz JR, Malviya S (1997). The FLACC: a behavioral scale for scoring postoperative pain in young children. *Pediatric Nursing*.

[92] Jay SM, Elliott C (1984). Behavioral observation scales for measuring children's distress: the effects of increased methodological rigor. *Journal of Consulting and Clinical Psychology*.

[93] Wheeler D, Vimalachandra D, Hodson EM, Roy LP, Smith G, Craig JC (2003). Antibiotics and surgery for vesicoureteric reflux: a meta-analysis of randomised controlled trials. *Archives of Disease in Childhood*.

[94] Oak SN, Kulkarni B, Chaubal N (1999). Color flow Doppler sonography: a reliable alternative to voiding cystourethrogram in the diagnosis of vesicoureteral reflux in children. *Urology*.

[95] Colodny AH, Lebowitz RL (1974). The importance of voiding during a cystourethrogram. *The Journal of Urology*.

[96] Arsanjani A, Alagiri M (2007). Identification of filling versus voiding reflux as predictor of clinical outcome. *Urology*.

[97] McGrath PA (1999). Commentary: psychological interventions for controlling children's pain: challenges for evidence-based medicine. *Journal of Pediatric Psychology*.

[98] Coté CJ (2001). “Conscious sedation”: time for this oxymoron to go away!. *Journal of Pediatrics*.

[99] Golparvar M, Saghaei M, Sajedi P, Razavi SS (2004). Paradoxical reaction following intravenous midazolam premedication in pediatric patients—a randomized placebo controlled trial of ketamine for rapid tranquilization. *Paediatric Anaesthesia*.

[100] Thurston TA, Williams CGA, Foshee SL (1996). Reversal of a paradoxical reaction to midazolam with flumazenil. *Anesthesia & Analgesia*.

[101] Sanders JC (2003). Flumazenil reverses a paradoxical reaction to intravenous midazolam in a child with uneventful prior exposure to midazolam. *Paediatric Anaesthesia*.

[102] Tucker CL, Slifer KJ, Dahlquist LM (2001). Reliability and validity of the brief behavioral distress scale: a measure of children's distress during invasive medical procedures. *Journal of Pediatric Psychology*.

[103] Wong DL, Baker CM (1988). Pain in children: comparison of assessment scales. *Pediatric Nursing*.

[104] Anderson BJ, Palmer GM (2006). Recent pharmacological advances in paediatric analgesics. *Biomedicine and Pharmacotherapy*.

[105] Herd DW, Salehi B (2006). Palatability of two forms of paracetamol (acetaminophen) suspension: a randomised trial. *Paediatric and Perinatal Drug Therapy*.

[106] Stevens B, Yamada J, Ohlsson A (2004). Sucrose for analgesia in newborn infants undergoing painful procedures. *Cochrane Database of Systematic Reviews*.

[107] Crellin D, Sullivan TP, Babl FE, O'Sullivan R, Hutchinson A (2007). Analysis of the validation of existing behavioral pain and distress scales for use in the procedural setting. *Paediatric Anaesthesia*.

[108] Borland M, Jacobs I, King B, O'Brien D (2007). A randomized controlled trial comparing intranasal fentanyl to intravenous morphine for managing acute pain in children in the emergency department. *Annals of Emergency Medicine*.

[109] Malinovsky J-M, Le Normand L, Lepage J-Y (1998). The urodynamic effects of intravenous opioids and ketoprofen in humans. *Anesthesia & Analgesia*.

[110] Malviya S, Voepel-Lewis T, Tait AR (1997). Adverse events and risk factors associated with the sedation of children by nonanesthesiologists. *Anesthesia & Analgesia*.

